# CT Coronary Angiography Studies After a Mean Follow-up of 3.8 Years in Children With Kawasaki Disease and Spontaneous Defervescence

**DOI:** 10.3389/fped.2020.00274

**Published:** 2020-05-28

**Authors:** Santosh Dusad, Manphool Singhal, Rakesh Kumar Pilania, Deepti Suri, Surjit Singh

**Affiliations:** ^1^Allergy Immunology Unit, Department of Pediatrics, Advanced Pediatrics Centre, Post Graduate Institute of Medical Education and Research, Chandigarh, India; ^2^Department of Radiodiagnosis and Imaging, Postgraduate Institute of Medical Education and Research, Chandigarh, India

**Keywords:** Kawasaki disease, spontaneous defervescence, dual source computed tomography coronary angiography, coronary artery abnormalities, 2D-echocardiography

## Abstract

**Background:** There is paucity of literature on follow-up of children with Kawasaki disease (KD) who have spontaneous defervescence during the acute stage and do not receive intravenous immunoglobulin. We report herein the role of computed tomography coronary angiography (CTCA) as an imaging modality in such situations.

**Methods:** This prospective observational study was carried out during the period January 2016–June 2017. Children underwent CTCA on 128-slice Dual Source CT (DSCT) scanner (Somatom Definition Flash, Siemens; Germany), and 2D-echocardiography on the same day.

**Results:** Mean age at time of diagnosis was 6.52 ± 3.13 years; range 2–14 years. Mean age at time of study was 11.03 ± 5.10 years; range 3.75–23.30 years. Mean interval between diagnosis of KD and time of present study was 3.84 ± 2.27 years. None of the patients showed any coronary artery abnormalities on either 2D-echocardiography or CTCA. While assessment of proximal segments of left main coronary artery, proximal right coronary artery, and left anterior descending artery was comparable on both 2D-echocardiography and CTCA, left circumflex artery, and distal right coronary artery could be clearly visualized only on CTCA.

**Conclusion:** In our experience, patients with KD who have spontaneous defervescence during the acute stage and do not receive IVIg may not have significant long-term coronary sequelae. CTCA is a useful imaging modality for delineation of coronary artery in patients with KD on long term follow-up especially in older children with thick chest walls and poor acoustic windows.

## Introduction

KD is an acute, systemic childhood vasculitis syndrome that mainly affects small children and has a predilection for coronary arteries. Diagnosis of KD is purely clinical and there are no laboratory tests available to confirm the diagnosis. Delays in diagnosis of patients with KD are not unusual and are more common in developing countries like India where awareness about this disorder is still low (1–6). Coronary artery abnormalities (CAAs) are known to develop in 15–25% of children with KD who do not receive appropriate and prompt treatment with IVIg. CAAs account for most of the morbidity and mortality associated with the disease (1, 7–9).

When a patient with KD presents in convalescent phase after spontaneous defervescence, decisions regarding initiation of treatment with intravenous immunoglobulin (IVIg) can be difficult and need to be individualized (1, 10). There is paucity of literature on long-term follow-up studies in children with KD who have had spontaneous defervescence. It was hitherto believed that patients with KD who have spontaneous defervescence tend to have a benign course (11, 12). However, recent studies have shown that KD patients with spontaneous defervescence may not always have milder phenotypes of the disease—in fact, the converse may well be true as incidence of CAAs in such situations may be higher than in patients who have been treated with IVIg (13, 14).

Echocardiography is considered to be the imaging modality of choice in acute stage as well as during follow-up of KD. Sensitivity and specificity rates of 95 and 99% have been reported for identification of coronary aneurysms by echocardiography (15). However, this imaging modality has several inherent limitations—these include poor visualization of middle and distal segments of coronaries, difficulties in visualization of left circumflex artery (LCx), chances of inter-observer variation, operator dependency, and poor acoustic windows in older children because of thick chest wall (12–14).

CTCA on dual source CT (DSCT) is a non-invasive tool that, under expert hands, has low radiation exposure and can accurately demonstrate CAAs in both proximal and distal coronaries. CTCA on DSCT has recently emerged as a non-invasive tool that has low radiation exposure and can accurately demonstrate the coronary wall anatomy and intraluminal abnormalities like thrombosis, stenosis, and calcification (16–18). There is, however, paucity of literature on CTCA performed during follow-up of children with KD who have had spontaneous defervescence.

We report herein the role of CTCA on a 128-slice DSCT platform in detecting CAAs in patients with KD who had spontaneous defervescence during the acute stage and did not receive IVIg for one or more reasons.

## Methods

This prospective observational study was carried out during the period January 2016–June 2017. Nineteen children (15 boys; four girls) with KD who had not received IVIg and were on regular follow up at Pediatric Allergy Immunology Unit, Advanced Pediatrics Centre, Postgraduate Institute of Medical Education and Research, Chandigarh, India were included in the study (19). Our institute is a not-for-profit federally funded tertiary care teaching hospital in North-West India.

Diagnosis of KD was based on diagnostic criteria given by AHA (4). These patients had not received IVIg because they either presented late in convalescent phase or had already became afebrile at time of presentation and had normal inflammatory markers. The study was approved by the Institute Ethics Committee, Institute Thesis Committee, and the Departmental Review Board. A written informed consent was obtained from the parents prior to enrolment. Children underwent CTCA on 128-slice Dual Source CT (DSCT) scanner (Somatom Definition Flash, Siemens; Germany), and 2D-echocardiography on the same day. The minimum and maximum dose length product (DLP) was 21 and 182 mGycm with mean DLP of 86.11 ± 48.02 mGycm. The scan time was 3.72 ± 0.60 s; range 2.85–4.76 s. Effective radiation dose was 1.19 ± 0.29 mSv; range 0.25–2.58 mSv. Diameters of major coronary arteries were recorded and coronary artery lesions, if any, were noted. Calcium scoring through DSCT was also carried out. Results of 2D-echocardiography that had been carried out at time of diagnosis were also taken into account. Seven patients had also undergone stress myocardial perfusion scintigraphy at initial diagnosis.

Findings of echocardiography were compared with those of CTCA. Paired sampled *t*-test was used to compare the average diameter of coronaries obtained by the two imaging modalities.

## Results

### Baseline Demographics

Mean age at time of diagnosis was 6.52 ± 3.13 years; range 2–14 years. Mean age of at time of study was 11.03 ± 5.10 years; range 3.75–23.30 years. The most common symptom present in this cohort of patients with KD was fever, that was present in all patients (100%). Mean duration of fever was 6.47 ± 5.44 days. Mean duration of symptoms before presentation to our institute was 21.79 ± 14.27 days. Sixteen patients (84.2%) were classified as incomplete KD. Principal clinical features included lip and oral cavity changes (31.6%), cervical lymphadenopathy in (52.6%), conjunctival injection (10.5%), polymorphous rash (52.6%), desquamation of fingers and/or toes (100%). Mean interval between diagnosis of KD and time of present study was 3.84 ± 2.27 years; range 2.4 months to 7.3 years. Seven patients had interval <2 years, 7 had interval between 2 and 5 years while five had interval more than 5 years (Table 1).

**Table 1:** Clinical profile of children with Kawasaki disease in the study (*n* = 19).

**Characterstics**	**Present**
Fever	19 (100%)
Rash	10 (52.6%)
Rash ≤ 1 week of fever onset	10 (100%)
Cervical lymphadenapathy	10 (52.6%)
Conjuctival injection	2 (10.5%)
Desquamation	19 (100%)
Lip and oral changes	6 (31.6%)

### Characteristics

Findings of echocardiography that had been carried out at initial admission were also taken into account. Prior to 2014 we had not been recording *Z* scores of coronary arteries. At that time, CAAs were categorized based on absolute internal diameter of vessels. Initial echocardiograms carried out at time of diagnosis showed normal coronary arteries in 16 of 19 children enrolled in the study. However, three children had CAAs—while two had mild dilatation of LMCA, one had irregular lumen of LAD with loss of distal tapering. All three patients had presented late to our institute in convalescent phase of disease and two of three had incomplete forms of KD. These changes had normalized on repeat echocardiography and CTCA performed at time of present study.

None of the patients in this series showed any CAA on either 2D-echocardiography or CTCA. While assessment of proximal segments of left main coronary artery (LMCA), proximal right coronary artery (RCA), and left anterior descending artery (LAD) was comparable on both 2DE and DSCT, left circumflex (LCx) and distal RCA could be clearly visualized only on CTCA.

Coronary artery diameters could be easily measured in all subjects by CTCA. However, on echocardiography LMCA diameter at the time of follow-up could be assessed accurately in 16, RCA and LAD in 15, and LCx in 11 subjects only (Table 2). All patients, except one, in whom coronary arteries were not visualized were aged more than 10 years and impaired visualization of coronary arteries was because of thick chest walls. Mean diameters of coronary artery measured by 2D-echocardiography and DSCT were comparable (Table 3).

**Table 2:** Mean coronary artery diameter by DSCT coornary angiography and 2D-echocardiography.

**Mean coronary artery diameter in mm**	**2D-Echocardiography**	**DSCT coronary angiography**
LMCA (Mean ±*SD*)	2.46 ± 0.47; (*n* = 16)	2.62 ± 0.46; (*n* = 19)
RCA (Mean ±*SD*)	2.14 ± 0.52; (*n* = 15)	2.28 ± 0.47; (*n* = 19)
LAD (Mean ±*SD*)	1.84 ± 0.31; (*n* = 15)	1.93 ± 0.28; (*n* = 18)
LCX (Mean ±*SD*)	1.46 ± 0.35; (*n* = 11)	1.70 ± 0.37; (*n* = 18)

*DSCT, Dual Source Computed tomography; LMCA, Left main coronary artery; RCA, Right coronary artery; LAD, Left anterior descending artery; LCx, Left circumflex coronary artery; SD, Standard Deviation*.

**Table 3:** Comparison between DSCT coronary angiography and 2D-echocardiography.

**Coronary artery**	**Mean ±*SD* in mm on DSCT Coronary angiography**	**Mean ±*SD* in mm on 2D-Echocardiography**	**Correlation**	***p*-value**
LMCA (*n* = 16)	2.54 ± 0.45	2.47 ± 0.47	0.77	0.36
RCA (*n* = 15)	2.25 ± 0.50	2.15 ± 0.53	0.92	0.08
LAD (*n* = 15)	1.89 ± 0.30	1.84 ± 0.31	0.50	0.51
LCX (*n* = 11)	1.56 ± 0.28	1.46 ± 0.35	0.76	0.18

*DSCT, Dual Source Computed tomography; LMCA, Left main coronary artery; RCA, Right coronary artery; LAD, Left anterior descending artery; LCx, Left circumflex coronary artery; SD, Standard Deviation*.

Stress myocardial perfusion scintigraphy was reported normal in six and showed mildly reduced perfusion defect in one child (20). Calcium scores were normal in all 19 patients.

## Discussion

The diagnosis of KD is always based on clinical criteria and there is no laboratory marker that can serve as a gold standard for diagnosis of this condition (21). A significant proportion of children with KD (25–50% in various series) have incomplete KD (7, 22–25). And this proportion would be higher in situations wherein the patients have reported late for diagnosis, as was the case in the present series (12, 13). Although 16/19 patients had incomplete KD at time of diagnosis, it is possible that some of the clinical features of KD may have disappeared by the time the children reported to us. Takahashi et al. have reported that incomplete forms of KD are more common in children who have had spontaneous defervescence as compared to those who have received IVIg (59.2 vs. 13.5%) (12). Hu et al. have also reported similar findings (13).

Delineation of coronary arteries in children with KD can be carried out by several imaging techniques. 2D-echocardiography is the preferred imaging modality both during the acute phase as well as during convalescence (1, 2, 4, 24, 26). However, there has been increasing interest about other imaging modalities that can address the limitations of echocardiography as described above. These include catheter angiography (CA), CTCA and magnetic resonance coronary angiography (MRCA) (16–18).

In this study we have assessed role of CTCA done on DSCT in evaluation of CAAs in patients with KD on follow-up. These children had had spontaneous defervescence during the acute stage and did not receive IVIg. Spontaneous defervescence is known to occur in a small proportion of patients with KD (1). CTCA is superior to 2DE for distal coronary artery visualization and for evaluation of the LCx. Further, assessment of the wall of coronary arteries is more accurate on CTCA than on 2DE. In older children, 2DE is often not very useful for evaluation of coronary arteries because of thick chest walls and the resultant poor acoustic window. In the present study, echocardiography could not visualize LCx and distal segments of coronary arteries in older children. CTCA accurately delineated the anatomy and lumen of coronary arteries and was especially useful for assessing distal segments of coronary arteries that are usually not visualized on 2DE.

Management of patients with KD and spontaneous defervescence is contentious and there is no consensus (1, 11, 12). Lin et al. had shown that regression of CAAs within 1 month of KD was more likely among patients who had been treated with IVIg compared to those who did not receive such treatment. However, authors have shown that late coronary outcome in these two group were comparable (11). Takahashi et al. retrospectively reviewed patients with KD who had had spontaneous defervescence. It was noted that there was no difference in development of CAAs between those that received IVIg therapy, and the ones who experienced spontaneous defervescence (12). Recently, Hu et al. have published data on outcomes of 37 children with KD who had spontaneous defervescence within 10 days. The incidence of CAAs at 1 month after disease onset was higher in patients with KD who had spontaneous defervescence as compared to those treated with IVIg (18.9 vs. 5.1%). Furthermore, it has been shown that new emergence of CAAs became significantly higher if they had not received IVIg therapy (due to spontaneous defervescence) when compared with those who did (13). These findings are a matter of serious concern and require further studies for confirmation.

CT calcium scoring is a technique that identifies calcium deposition in coronary arteries without the need of intravenous contrast. Calcium deposition is considered a surrogate marker for previous coronary artery involvement in patients with KD. Kahn et al. have shown that calcium deposition was not seen in patients with KD who did not develop CAAs during acute phase of disease (27). The authors have shown that calcium scoring by CT is also a useful tool for identification of unrecognized CAAs in patients with a remote history of KD. In the present study we have also performed calcium scoring and did not find any abnormalities. Our findings are in consonance with the results of Kahn et al. (27).

To conclude, this study shows that patients with KD who had spontaneous defervescence did not develop any significant long-term coronary sequelae. This is reassuring. The strengths of our study are that all diagnoses were confirmed by one individual (the senior author—SS) and there was uniformity of data collection as this was a single center cohort. Further, CTCAs were performed under direct supervision of the consultant radiologist (MS) in all cases using radiation optimized protocols. The number of patients included in the study is undoubtedly small. However, this is due to the fact that we have only enrolled children with KD who had spontaneous defervescence during the acute stage and did not receive IVIg. In our experience CTCA is a useful imaging modality for delineation of coronary arteries in patients with KD on long term follow-up, especially in older children who have thick chest walls and poor acoustic windows. Further long-term and multicentric large cohort studies are warranted on this aspect of KD.

## Data Availability Statement

All datasets generated for this study are included in the article/supplementary material.

## Ethics Statement

The studies involving human participants were reviewed and approved by Institute Ethics Committee, Postgraduate Institute of Medical Education and Research, Chandigarh, India.

## Author Contributions

SD, MS, and RP wrote the initial draft of manuscript and were also involved in editing and revision of manuscript at all stages of its production. SD and RP collected data, performed statistical calculations, analyzed, and critically interpreted the results. DS reviewed the literature and contributed to editing of manuscript. SS conceived the idea, reviewed the literature, critically reviewed and edited the manuscript at all stages of its production. All authors discussed the results and read and approved the final manuscript.

## Conflict of Interest

The authors declare that the research was conducted in the absence of any commercial or financial relationships that could be construed as a potential conflict of interest.
